# Chin tuck against resistance exercise with feedback to improve swallowing, eating and drinking in frail older people admitted to hospital with pneumonia: protocol for a feasibility randomised controlled study

**DOI:** 10.1186/s40814-022-01060-w

**Published:** 2022-05-19

**Authors:** David G. Smithard, Ian Swaine, Salma Ayis, Alberto Gambaruto, Aoife Stone-Ghariani, Dharinee Hansjee, Stefan T. Kulnik, Peter Kyberd, Elizabeth Lloyd-Dehler, William Oliff

**Affiliations:** 1 Queen Elizabeth Hospital, Lewisham and Greenwich NHS Trust, Stadium Road, SE18 4QH Woolwich, UK; 2 University of Greenwich, Avery Hill Campus, Avery Hill Rd., London, SE9 2BT UK; 3 King’s College London, Department of Population Health Sciences, Faculty of Life Sciences & Medicine, Guy’s Campus, London, SE1 1UL UK; 4 Department of Mechanical Engineering, University of Bristol, Bristol, BS8 1TH UK; 5 University of Greenwich, Medway Campus, Central Avenue, Gillingham, ME4 4TB UK; 6 Ludwig Boltzmann Institute for Digital Health and Prevention, Lindhofstrasse 22, 5020 Salzburg, Austria; 7 School of Energy and Electronic Engineering, University of Portsmouth, Anglesea Building, Anglesea Road, Portsmouth, PO1 3DJ UK; 8 Independent Service User Representation, London, UK; 9 University of Greenwich, Old Royal Naval College, Park Row, London, SE10 9LS UK

**Keywords:** Chin tuck against resistance, Dysphagia, Frailty, Old age, Rehabilitation, Swallowing

## Abstract

**Background:**

Swallowing difficulties (dysphagia) and community-acquired pneumonia are common in frail older people and maybe addressed through targeted training of the anterior neck musculature that affects the swallow. We have developed a swallowing exercise rehabilitation intervention (CTAR-SwiFt) by adapting a previously established swallowing exercise to ensure patient safety and ease of execution in the frail elderly population. The CTAR-SwiFt intervention consists of a feedback-enabled exercise ball that can be squeezed under the chin, with real-time feedback provided via a mobile application. The aim of this study is to evaluate the feasibility of assessing the effectiveness of the CTAR-SwiFt intervention in reducing dysphagia and community-acquired pneumonia, prior to a larger-scale multi-centre randomised controlled trial.

**Methods:**

We will recruit 60 medically stable patients over the age of 75 years who have been admitted with a diagnosis of pneumonia to the acute frailty wards at two participating hospitals in the UK. Study participants will be randomised into one of three groups: standard care, low intensity (once daily) CTAR-SwiFt exercise or high intensity (twice daily) CTAR-SwiFt exercises. The intervention period will last for 12 weeks, the final follow-up assessment will be conducted at 24 weeks. We will assess the feasibility outcomes, including rates of participant recruitment and retention, compliance with the exercise regime and adverse incidents. Additionally, we will assess the usability and acceptability of the intervention device and the performance of different clinical outcome measures (e.g. chin tuck strength, Functional Oral Intake Scale, SWAL-QOL, EQ-5D and swallow speed). A sub-sample of study participants will complete videofluoroscopic assessments of swallowing function before and after the intervention to evaluate the physiological changes (e.g. bolus flow rates, laryngeal elevation, base-of-tongue retraction).

**Conclusions:**

By improving the ability to swallow, using our chin tuck exercise intervention, in frail older patients admitted to hospital with pneumonia, it is anticipated that patients’ oral intake will improve. It is suggested that this will further impact clinical, patient and healthcare economic outcomes, i.e. reduce the need for supplemental feeding, improve patient satisfaction with oral intake and swallowing-related quality of life, decrease the occurrence of chest infections and reduce hospital admissions and related healthcare costs.

**Trial registration:**

ISRCTN, ISRCTN12813363. Registered on 20 January 2020

**Supplementary Information:**

The online version contains supplementary material available at 10.1186/s40814-022-01060-w.

## Background

Many (55%) older frail people admitted to hospital will have difficulties with swallowing (dysphagia) [1]. Up to 30% of older people living at home may have dysphagia [2], and 28% of older people are identified as aspirating saliva on instrumentation [3, 4]. In those admitted with a diagnosis of community-acquired pneumonia, up to 90% may have aspirated saliva or food. With age, there is an increased risk of aspiration due to changes in motor function, which is often subtly compensated for [5, 6]. There is a possibility that dysphagia is more common than the published data suggest, because many older people do not report problems [6] or have learnt to live with them [5].

With age, there is increased residue remaining after the swallow [6] secondary to reduced opening and higher resting pressures off the upper esophageal sphincter (UES) [7] with an increased dwell time of the bolus in the pharynx; these changes correlate well with the known reduction in laryngeal elevation with age [7]. Weakness in the supra-hyoid muscles (such as that induced by sarcopenia), weakness of the laryngeal elevation and anterior motion, reduced epiglottis depression and reduced opening of the UES all contribute to dysphagia in frail older people [7, 8].

Despite the high frequency of dysphagia, swallowing problems/dysphagia in frail older people is poorly managed in hospitals. Swallowing is not routinely assessed when frail people are admitted to the hospital as occurs with acute stroke patients [9, 10], and dysphagia is not always identified. As a consequence, rehabilitation of the swallow is not provided. Momosaki et al. [11] using a large Japanese database demonstrated that those patients with dysphagia who were offered appropriate rehabilitation were more likely to have a total oral intake compared to those not offered oral-pharyngeal rehabilitation (OR 1.3, *p* < 0.001).

Changes in crico-pharyngeal distensibility or traction, generated by suprahyoid muscle contraction, will result in dysphagia [8]. Yet, standard hospital rehabilitation frequently consists of postural manoeuvres (including chin tuck) to enable a safe swallow rather than an improved swallow. It would seem logical that an approach to swallow rehabilitation would be to improve the strength of the suprahyoid muscles. Skeletal (arm and leg) muscle weakness, as treated with resistance exercise, has been investigated and shown to have a positive effect on muscle strength and bulk [12]. Resistance exercises may prevent loss or improve muscle bulk and strength [13–15].

We have worked with a group of patients (patient and public involvement in research) [16] who have experienced dysphagia, and they expressed the need for better rehabilitation of swallowing after dysphagia has been diagnosed. With the help of the patients, we have developed a swallowing exercise rehabilitation intervention (CTAR-SwiFt), by modifying the previously established Shaker swallowing exercise [17] and making it safer and easier to use. This was achieved by introducing a simple feedback-enabled exercise ball that can be squeezed under the chin.

There are many unknowns with respect to swallowing physiology and the anatomical structures that are involved in the CTAR-SwiFt intervention and how this swallowing exercise results in an improvement in swallowing and a reduction of aspiration. With active swallow rehabilitation, the appropriate dose of exercise required for benefit is not known, i.e. how frequently the exercise should be undertaken, how many repetitions should be performed per session and at what force/pressure change the exercise should be carried out. These parameters are required for patient benefit and optimal compliance with an exercise rehabilitation intervention.

## Methods

This protocol follows the Standard Protocol Items: Recommendations for Interventional Trials (SPIRIT) [18] and the Consolidated Standards of Reporting Trials (CONSORT) extension for randomised pilot and feasibility trials [19] reporting standards, as recommended for protocols of pilot and feasibility studies [20]. The SPIRIT checklist is provided in Additional file 1.

### Aim and objectives

The aim of this study is to evaluate the feasibility of assessing the effectiveness of the CTAR-SwiFt intervention in reducing dysphagia and community-acquired pneumonia, prior to a larger-scale multi-centre randomised controlled trial.

The following primary objectives are to:

- Establish whether it is feasible to recruit enough participants
- Assess the recruitment rates across each of the two acute hospital recruitment sites
- Assess the willingness of participants to participate in and complete the intervention
- Assess the compliance with the home-based daily exercise programme
- Establish the measurement variability of the tools for assessing outcome, e.g. Functional Oral Intake Scale (FOIS), quality of life (QoL, SWAL-QOL, EQ-5D) and swallow speed
- Assess the ease of use and acceptability of the intervention (including the CTAR-SwiFt feedback ball)
- Determine whether patients are willing and able to undergo VF
- Identify the optimum dose of CTAR-SwiFt training (daily vs twice daily frequency)

The following secondary objectives are physiological changes as measured using VF:

- Assess changes in bolus flow rates
- Measure the percentage change in laryngeal elevation
- Measure the percentage change in base-of-tongue retraction during a swallow
- Establish whether there is a reduction in the pharyngeal residue after the intervention
- Observe the timing of UES opening, before and after the intervention

### Design and setting of the study

The study is a randomised controlled feasibility study. We aim to recruit 60 patients [21–23], age 75 years or more, to be randomised into one of three groups:

1. Usual standard care (as defined by the clinical team including the speech and language therapist)
2. Usual care + low-intensity rehabilitation (once daily CTAR-SwiFt exercises)
3. Usual care + high-intensity rehabilitation (twice daily CTAR-SwiFt exercises)

The study will be undertaken at two acute hospital sites in the UK: Queen Elizabeth Hospital, Woolwich, and Southmead Hospital, Bristol. Participants will be identified from those admitted acutely to the hospital.

### Participant eligibility criteria

The inclusion criteria are as follows:

- Admitted with a diagnosis of pneumonia
- Medically stable: systolic BP > 110, heart rate > 60 bpm and Modified Early Warning Score (MEWS) ≤ 1
- Over the age of 75 years (though someone fulfilling the frailty criteria who is slightly below this age will be considered)
- Clinical Frailty Score of ≥ 4 and < 8
- Montreal Cognitive Assessment (MOCA) > 14
- Able to provide consent (different media will be provided to patients to enable consent to occur, e.g. pictures, speech and language therapy support)
- No significant breathlessness (St George’s COPD Score)
- Not requiring oxygen
- No past history of stroke or neurological disease
- No evidence of severe rheumatoid arthritis (risk of neck instability)

The exclusion criteria are as follows:

- Failure to provide consent to take part in the study
- Progressive medical conditions (e.g. due to malignancy or progressive neurological disease)
- MOCA < 14
- Dysphagia requiring active intervention at the time of assessment
- Dysphagia secondary to surgical treatment of head and neck cancer

### Recruitment

All consecutive patients will be identified and screened in the acute frailty wards of the participating hospitals. The responsible medical team will identify the patients to the research team, who will be based in the ward. Identified patients will be screened against study eligibility criteria by the research team, prior to consent being sought.

Consent will be obtained by either the research staff or the responsible medical consultant. All patients participating in the study will be asked to provide informed consent. A patient information sheet will be provided prior to consent being sought. Participants will be given at least 48 h to consider whether they wish to be involved in the study.

### Randomisation

Patients will be randomly allocated to one of the 3 study groups using a web-based randomisation system provided by the Clinical Trials Unit at King’s College London College London (KCTU). Randomisation will be at the level of the individual, using the method of stratified block randomisation, with randomly varying block sizes.

Participant initials and date of birth will be entered into the randomisation system. No data will be entered into the randomisation system unless a participant has signed a consent form to participate in the trial. Randomisation will be undertaken centrally by the coordinating study team, by authorised staff logging onto the randomisation system, thus ensuring allocation concealment. A full audit trail of data entry will be automatically dated and time-stamped, alongside information about the user making the entry within the system.

### Study assessments

Data collection will follow the study protocol that provides a detailed description of the type of data to be collected and the timing of data collection. The sequence of study procedures and assessments is shown in Table 1.

**Table 1:**
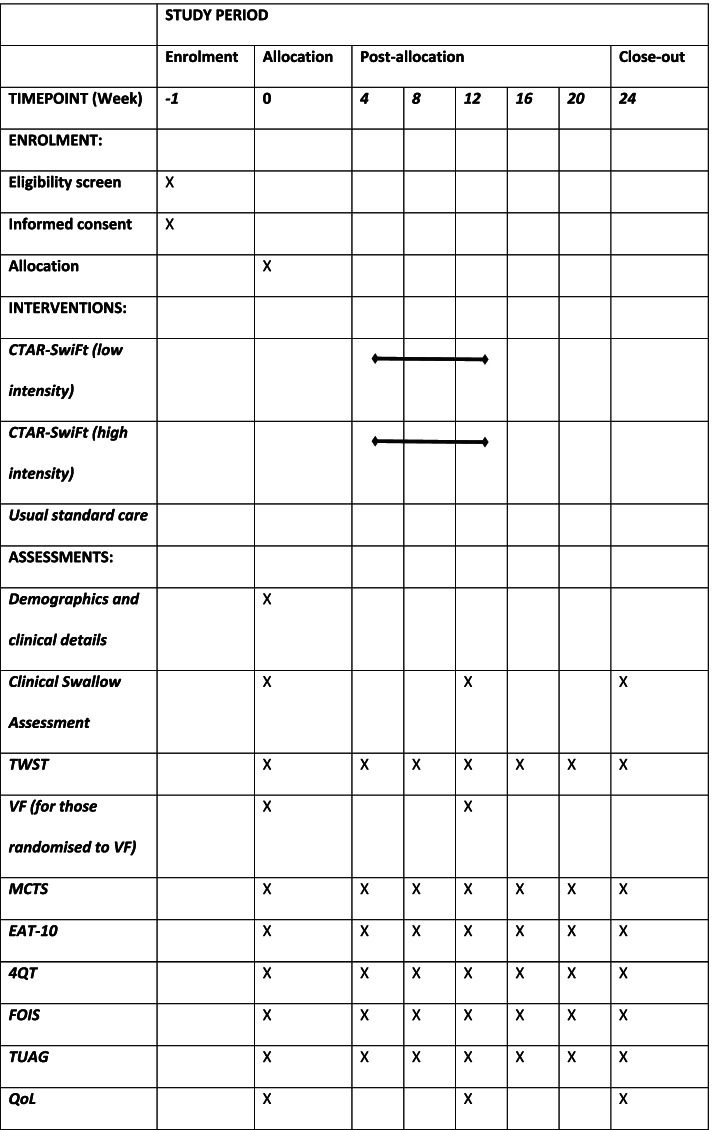
Study procedures (SPIRIT figure) [18]

*EAT-10* Eating Assessment Tool, *FOIS* Functional Oral Intake Scale, *MCTS* maximal chin tuck strength, *QoL* quality of life, *TUAG* Timed Up and Go, *TWST* Timed Water Swallow Test, *VF* videofluoroscopy

#### Baseline assessments

Baseline data collection includes the following:

- Age
- Sex
- Co-morbidities which may affect the ability to use the CTAR-SwiFt feedback ball (e.g. severe rheumatoid arthritis)
- Medication use
- Swallowing assessments
- Assessments of study outcome parameters (Table 1)

#### Clinical swallowing assessment

Each participant will undergo a standardised clinical swallowing assessment by a speech and language therapist, and if clinically indicated, advice on the management of dysphagia and swallowing will be offered. Those people where the presence of dysphagia is pre-existing or where there is a clinical concern will be excluded from the study.

#### Videofluoroscopy

Instrumental assessment of swallowing is undertaken using either fibreoptic endoscopic evaluation of swallowing (FEES) or videofluoroscopy (VF). These two evaluations are complimentary and on occasions are used in tandem. VF has the advantage of being able to demonstrate physiology and function at the same time, in both the anterior-posterior and lateral projections if required. Studies of pharyngeal function for swallowing manoeuvres such as chin tuck have used VF [24, 25].

A sub-group of thirty participants will be randomly drawn from the intervention arms and undergo VF (pre- and post-intervention). VF will be conducted by a speech and language therapist and a radiographer following standard clinical procedures and protocols, in the lateral plane with the exposure field set between the lips and the back of the neck.

Six swallows will be assessed using three consistencies twice. Of the two swallows per consistency, one will be performed whilst using the CTAR-SwiFt feedback ball and one without. The timing of the transit of the various consistencies will be measured [26]. Additionally, the sequence of fluoroscopic images will be processed in order to extract the motion of the passage walls as well as the bolus. VF investigations address the secondary study objectives as listed above.

#### Timed Water Swallow Test

The Timed Water Swallow Test is a simple assessment of swallowing speed. The participant will be provided with 90 ml of water to drink. They will be timed as to how long it takes to comfortably drink the 90 ml and how many sips were taken. If the total volume was not drunk, the residual will be recorded.

#### Questionnaires and functional assessments

The following are the questionnaires and functional assessments:

- EAT-10 is a validated questionnaire swallow screen. There are 10 variables with scores of 0–4. A score of > 3 is indicative of dysphagia [27].
- 4QT is a simple 4-question swallow screening tool [28].
- QoL will be assessed using the EQ-5D [29] and SWAL-QOL [30].
- FOIS is a functional score of the amount that can be eaten, type and consistency. It is scored between 1 (unable to eat or drink) and 7 (normal diet) [31].
- Timed Up and Go (TUAG) measures the time taken to rise from a chair, walk 3 m, turn around and sit back down [32].

#### Qualitative assessments

Semi-structured qualitative interviews with study participants will be conducted to determine key issues of concern for participants [33]. Twelve participants (20% of the entire sample) will be purposively recruited, to represent participants from all 3 study arms, even gender split, old and very old participants, those with good exercise completion and those with low exercise completion and those with informal care support at home and those without. This should be able to provide data covering all relevant points to enable the research team to understand how a phenomenon is seen and understood among different people, in different settings and at different times. Qualitative data will be transcribed and analysed thematically, allowing the research team to understand participants’ experiences of study participation and identification of common and variable points [34].

#### Feedback questionnaires

All participants will be provided with a feedback questionnaire. The questionnaire will ask about the organisation of the study and how it could have been ‘run better’.

Participants will be approached to provide their views on the CTAR-SwiFt feedback ball, instructions on its use and ease of application. This will be conducted by survey with closed and open questions.

#### Assessment of compliance with treatment

The investigational device includes software which will record compliance, i.e. the frequency and nature of the chin tuck exercises actually undertaken by the participant. This will be compared with the recommended allocated exercise sessions provided at the outset of the programme.

#### Safety/adverse events

Any adverse event (clinical or device-related) will be reported to the study co-ordinator and/or chief investigator according to Good Clinical Practice (GCP) and using a standard reporting form. A description of the adverse event and the outcome to the event will be recorded.

### Intervention

The CTAR-SwiFt intervention is CTAR performed using an air-filled bladder (typically a small ball) with a solid-state electronic battery-powered pressure gauge that connects to an Android smartphone or tablet by Bluetooth, giving feedback on exerted pressure/effort to target the level most appropriate for the individual participant.

#### Investigational device

With the help of patients, clinicians and therapists, we have developed a simple chin tuck feedback-enabled exercise, which works by squeezing a small rugby ball which is placed under the chin, in an arrangement that was originally used by Yoon et al. [35]. However, our exercise ball allows the level of pressure that is exerted during the chin tuck squeeze to be monitored by a small pressure gauge. This exertional pressure is transmitted to a monitoring and display device (Android smartphone or tablet), which provides visual feedback to the participant. By adjusting the level of effort exerted in squeezing the ball, the participant can match their effort with a predetermined safe ‘target’ (which is determined by the clinician and researchers). This feedback system thereby ensures that repeated periods of chin tuck exercise are performed by the patient at a safe and consistent effort level each time.

#### Intervention procedures

**Fig. 1:**
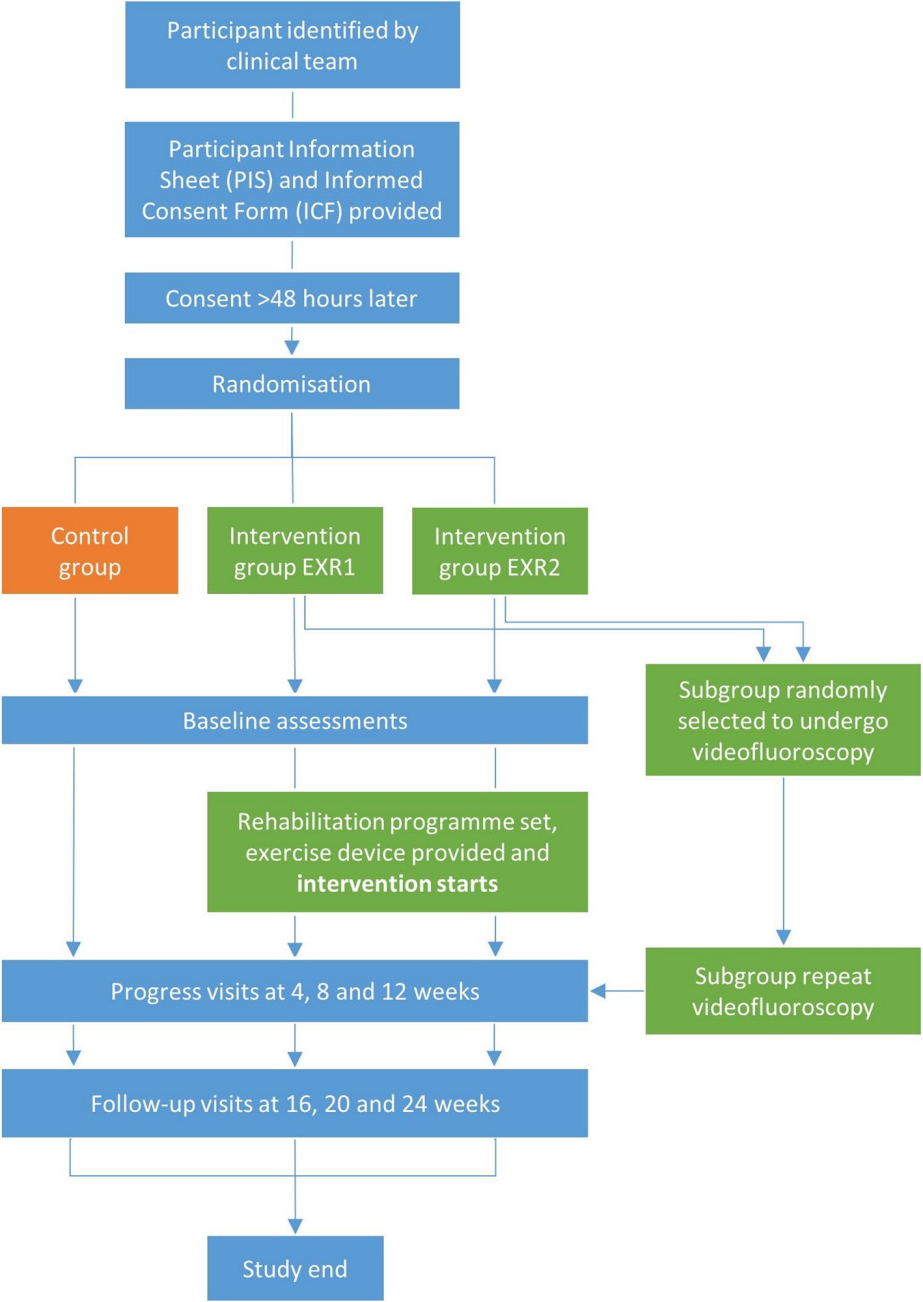
Study flow chart

Patients will be randomly allocated to a lower or a higher intensity intervention group: exercise rehabilitation ‘once-per-day’ group (EXR1) or ‘twice-per-day’ group (EXR2, Fig. 1). Each participant who is allocated to either of the intervention groups will be asked to perform CTAR using the investigational device.

- One exercise session, at monthly intervals (for the intervention arms) will be performed in a supervised way, at the Queen Elizabeth Hospital and Bristol Northern Hospital, and the other daily exercise sessions will be performed at home.
- At each monthly supervised session, an assessment of maximum chin tuck strength (MCTS) will be performed by the speech and language therapist. This will involve 3× chin tuck exercises for 3–5 s during which the participant will be asked to exert as much chin tuck force against the ball as possible.
- In the intervention groups, the MCTS value will be used to determine an individual safe submaximal target intensity (effort level) for the CTAR-SwiFt exercise, which will usually be set at 30% of MCTS.
- When performing the CTAR-SwiFt exercise, patients will be asked to use the visual feedback provided on the smartphone or tablet to adjust their effort until it matches the individual target intensity (effort level, set by the speech and language therapist). Patients will be asked to maintain this effort until the specified time period (i.e. 1 min) has elapsed.
- Patients will be asked to repeat these 1-min CTAR exercise periods, three times, and there will be 1 min of rest in between each.

Each participant will then be asked to complete (at home) either one (EXR1 group) or two (EXR2 group) sessions of CTAR-SwiFt exercises per day, for 12 weeks, in the format established during the previous supervised hospital-based exercise session. The overall ease with which each exercise session is completed will be assessed by asking patients to complete a simple exercise ‘comfort rating scale’ (CR-10) [36] after every single session. The ease of completion rating will be used by the patient and clinic staff, to ensure that the exercises do not require excessive effort from patients. The effort level should be maintained at 30% of MCTS (as assessed in the monthly supervised sessions). The rationale for this is as follows: Although the intention is for the effort level to remain constant at 30% of MCTS, as chin tuck strength increases during the intervention, the muscular force required to maintain 30% of MCTS will also increase. It is therefore anticipated that it may be necessary to reduce the target from 30 to 25% of MCTS in some patients, as the intervention progresses.

### Control condition

One-third of the participants will be randomised to the control condition, which consists of the usual treatment (Fig. 1). Participants in the control group will undergo monthly MCTS assessments as participants in the intervention groups but will not conduct daily CTAR-SwiFt exercises.

### Outcomes

Outcomes include feasibility and acceptability outcomes to inform the feasibility of conducting a definitive trial, as well as relevant clinical outcomes for future prospective sample size calculation for a definitive trial:

1. Recruitment rates across two NHS hospital sites assessed using the number recruited per month and the total in the study period, recorded using trial management software.
2. Willingness to be recruited to the study assessed using the number of people who refuse and their reasons for refusing, recorded using trial management software.
3. Study retention (< 30% drop out) assessed using the number not completing the study relative to the number agreeing to participate, recorded using trial management software.
4. Compliance: 80% of exercises undertaken (daily percentage averaged over the intervention period), monitored via the CTAR-SwiFt Android application which records exercise data for all sessions completed.
5. The absence of adverse incidents assessed, using the number of reports recorded in trial management software.
6. Acceptability of intervention, assessed using a 5-point questionnaire.
7. Mechanics of swallowing, assessed using VF (reduced pharyngeal transit time, reduced residue post swallow).
8. Aspiration prevention, assessed by analysing laryngeal movement, UES opening and tongue base retraction as visualised on VF.
9. Swallow speed assessed using the Timed Water Swallow Test (TWST; time taken to drink 90 ml of water).
10. Ease of use of the CTAR-SwiFt feedback ball, assessed using verbal feedback and a usability questionnaire.
11. Swallowing ability assessed using FOIS, as completed by the medical staff.
12. Dysphagia-related QoL, assessed using the SWAL-QOL questionnaire.
13. Health-related QoL, assessed using the EQ-5D questionnaire.
14. Strength and mobility assessed using the TUAG test. Participants must stand from sitting, walk 3 m, then turn around, walk back to the chair and sit down again.

Study assessments are conducted monthly, with all measures being completed at baseline, 12 weeks and 24 weeks (Table 1).

### Sample size justification

This is a feasibility study, and as such, it is not powered to detect a statistically significant intervention effect. Sixty participants will be recruited, which was considered to be adequate to identify the possibility of progressing to a larger definitive study and is a generally recommended sample size for feasibility studies [21]. The objective measures, process mapping and qualitative data collected will aid in that decision.

The study is aiming to recruit 60 patients, at a minimum recruitment rate of 2 participants per site per month.

### Analysis

#### Feasibility and clinical outcomes

On the completion of the study, the data will be cleaned and analysed. Recruitment rate, retention and attrition are simple percentages of those recruited. The feasibility criteria (willingness to be recruited to the study, study retention (< 30% drop out), compliance (80%, daily percentage of exercises undertaken averaged over the intervention period), absence of adverse incidents and acceptability of intervention) will be used to establish whether it is feasible to progress to a larger-scale multi-centre randomised controlled trial. There will be comparisons between the three arms of the study. Non-parametric tests will be utilised to compare between the groups. Improvements in FOIS, TWST and TUAG will use parametric tests to compare between-group and within-subject changes.

#### Videofluoroscopy

VF data will be analysed in two different ways: (1) The physiological effects of the chin tuck exercise rehabilitation intervention on the base of the tongue, posterior pharyngeal wall and laryngeal movement have not been fully clarified. Therefore, we will examine the changes in the movements of these three structures, with and without CTAR. (2) This data will be used as input to computational fluid dynamics simulations in order to determine the precise values of pressure, velocity, efficiency of bolus mixing and the stress exerted on the passage walls by the transiting bolus. This will establish the quantitative measures that will be important in establishing the repeatability of swallows and in evaluating the efficacy of the chin tick exercise.

## Discussion

A number of previous research studies have examined the interventions which aim to rehabilitate swallowing function through targeted strengthening of the suprahyoid muscles. The CTAR-SwiFt intervention builds on this literature, but also adds important novel aspects.

A swallow programme including strengthening exercises (Swallow-STRONG) which consist of tongue strengthening exercises [37] and the Shaker manoeuvre (neck exercises) [17] have been shown to improve swallow mechanics. Shaker and colleagues developed a system of ‘head raising’ exercises to strengthen the hyoid group of muscles and neck muscles [17]. The exercise programme is conducted in a supine position and consists of 3 head-raising exercises, held for 60 s each, followed by rapid neck flexion, for 30 times daily for 6 weeks. These exercises also involve activation of the anterior neck muscles (sternocleidomastoid) and abdominal muscles not directly related to swallowing. The exercise programme strengthens the suprahyoid muscles resulting in the improved upper esophageal opening (*p* < 0.01), laryngeal anterior excursion (*p* < 0.05) and reduction in post-swallow aspiration (*p* < 0.05) [17].

Mapani et al. [38] found that the Shaker exercise resulted in an increase in thyrohyoid shortening after 6 weeks compared to tongue exercises and swallowing manoeuvres. Some studies suggest that this type of exercise causes increased contraction pressure in the pharynx, increased pressures in the pyriform sinuses and shortened opening times of the UES. However, this is in contrast to some other studies [39, 40].

In frail older people, the Shaker exercise may not be technically feasible due to muscle weakness, fatigue or co-existing morbidities. Sze et al. [8] showed that it was possible to do this type of exercise by placing a partially inflated ball beneath the chin and then pressing down against the ball. This exercise—chin tuck against resistance (CTAR)—generated similar electromyography results and greater benefits than the Shaker exercise [35, 40]. Recently, Shaker and colleagues have suggested an alternative approach—‘laryngeal resistance’ [41].

The chin tuck is a swallowing exercise that is often deployed where the swallowing problem is secondary to a delay in the onset of the pharyngeal swallow. It is generally accepted that the exercise pulls the larynx up and forwards and at the same time opens the UES [17, 42]. Such a system is being utilised as part of the Ampcare neuromuscular stimulation programme [43, 44]. However, at present, it is not possible to perform the chin tuck exercise in a consistent way, with controlled effort because there is no means by which effort can be regulated by the patient. Moreover, Balou et al. [39] suggest that more data is required to determine how chin tuck affects the physiology of swallowing. The advantage of the chin tuck exercise, over the Shaker movement, is that the effects are more localised and less likely to unnecessarily recruit the large anterior muscles of the neck. Park et al. [45] studied stroke patients and showed positive clinical benefits of CTAR in a small randomised study.

Rogus-Pulia et al. [37] used isometric progressive resistance oropharyngeal therapy to demonstrate improved FOIS score (effect estimate 0.4, *p* < 0.02), reduced incidence of pneumonia and reduced number of hospital admissions, in a cohort that was mixed in aetiology of dysphagia. However, previous exercise interventions have not been carefully controlled. The ability to control one’s own effort during rehabilitation is essential in order to undertake the exercise in a consistent and regulated way, especially if it is to be performed by the patient at home. No exercise rehabilitation devices provide feedback that allows the patient to carefully control their effort when exercising at home. Furthermore, no previous exercise programmes allow logging of exercise data for subsequent review by the therapist in the clinic. The real-time feedback on exercise performance and the objective automated recording of completed exercises are important novel aspects of the CTAR-SwiFt swallowing exercise intervention.

As has been the case for much clinical research that is unrelated to coronavirus disease 2019 (COVID-19) [46], the COVID-19 pandemic has impacted the planning and conduct of this study also. Clinical services at the recruiting sites have continued with all non-elective care. Recruitment has therefore been impacted by the acuity of the presentation of the patients who have presented to and been admitted to hospital, resulting in a reduction in the expected patient population for the study. Patients who present with COVID-19 pneumonitis, a large proportion of admissions during the pandemic, are excluded from the study. Over the pandemic period, patients have had more rapid discharges from the hospital, resulting in a shorter period when patients who meet the study eligibility criteria are in hospital, and therefore, some suitable participants cannot be approached by the research team prior to discharge.

All follow-up appointments are taking place in the community or via telehealth (as opposed to initial plans of follow-up in a hospital outpatient clinic). The research team contact patients when their follow-up visits are due and ask whether they would prefer a home visit or telephone consultation. It should be noted that telephone consultations impact the data collection of the outcome measures TWTS and TUAG. Whilst these measures could be completed over a video call, the participants of this study have anecdotally expressed low levels of confidence using video calls. The timing of follow-up visits has also been impacted by researchers, patients or their families contracting COVID-19 or having to self-isolate.

Contingency plans include reduced recruitment target to the VF sub-group, as this requires attendance at the hospital for elective imaging. As VF can only be carried out in the hospital, this may also increase participant risk to exposure to COVID-19. A personal protective equipment (PPE) protocol has also been put in place for face-to-face follow-up visits, with researchers required to comply with the hospital PPE policy.

Future plans to continue this research will be based on findings from this study. A decision to proceed to a prospectively powered, definitive randomised controlled trial will be made according to feasibility outcomes, including recruitment and retention of study participants, participant compliance and acceptance of the CTAR-SwiFt intervention, and evidence of safety and potential effectiveness of the intervention. Aspects of the CTAR-SwiFt intervention and the trial design may be revisited according to learnings from this study. For example, it is acknowledged that this study protocol does not include any direct assessments of sarcopenia, such as imaging-based or invasive assessments of muscle mass, which were considered too burdensome for participants. The indicators that will allow the comparison of sarcopenia/frailty between the study groups are the Clinical Frailty Score, which is recorded as part of the eligibility screen, and the TUAG test. TUAG is a widely used measure in older clinical populations which incorporates lower limb strength (rising from the chair), gait speed and balance (180-degree turns), and it has been recommended for clinical assessment of sarcopenia [47]. Other similarly convenient and recommended clinical measures of sarcopenia, in particular, hand grip strength and anthropometric measurements such as calf and mid-upper arm circumference [47], may be considered in the design of a follow-up study.

## Conclusion

This study aims to evaluate the feasibility of conducting a definitive large-scale multi-centre trial of the CTAR-SwiFt intervention, a novel approach to exercise rehabilitation of the swallow using a feedback-enabled ball in combination with an Android application for smartphone or tablet. By improving the ability to swallow in frail older patients admitted to hospital with pneumonia, it is anticipated that patients’ oral intake will improve. It is suggested that this will further impact clinical, patient and healthcare economic outcomes, i.e. reduce the need for supplemental feeding, improve patient satisfaction with oral intake and swallowing-related quality of life, decrease the occurrence of chest infections and reduce hospital admissions and related healthcare costs.

## Trial status

Following delays due to the global coronavirus disease 2019 (COVID-19) pandemic, recruitment to the study started on 08 June 2021. Anticipated recruitment end date is 03 November 2022.

## Supplementary Information

**Additional file 1.** SPIRIT Checklist.

## Data Availability

Not applicable.

## Abbreviations

CTAR: Chin tuck against resistance
CTAR-SwiFt: Intervention under investigation in this study
CR-10: Comfort Rating Scale
EAT-10: Eating Assessment Tool
EQ-5D: EuroQuol Health-Related Quality of Life Questionnaire
FEES: Fibreoptic endoscopic evaluation of swallowing
FOIS: Functional Oral Intake Scale
GCP: Good Clinical Practice
KCTU: King’s Clinical Trials Unit
MCTS: Maximum chin tuck strength
MEWS: Modified Early Warning Score
MOCA: Montreal Cognitive Assessment
PPE: Personal protective equipment
SWAL-QOL: Swallowing Quality of Life Questionnaire
TUAG: Timed Up and Go
TWST: Timed Water Swallow Test
UES: Upper esophageal sphincter
VF: Videofluoroscopy
